# [^99^ᵐTc]Tc-PSMA-I&S SPECT/CT quantitative parameters for risk stratification and metastasis prediction in primary prostate cancer: a retrospective study

**DOI:** 10.3389/fmed.2025.1654685

**Published:** 2025-09-30

**Authors:** Ming Li, Zhenglian Gao, Jiangming Sun, Xiangyu Li, Changping Liang, Tao He

**Affiliations:** ^1^Department of Nuclear Medicine, Panzhihua Central Hospital, Panzhihua, Sichuan, China; ^2^Nuclear Medicine and Molecular Imaging Key Laboratory of Panzhihua, Panzhihua, Sichuan, China; ^3^Department of Anesthesiology, Panzhihua Central Hospital, Panzhihua, Sichuan, China

**Keywords:** prostate cancer, ^99m^Tc-PSMA-I&S, single-photon emission computed tomography, standardized uptake value, risk assessment

## Abstract

**Background:**

To evaluate the diagnostic performance of [^99^ᵐTc]Tc-PSMA-I&S SPECT/CT in primary prostate cancer (PCa) detection and assess its ability to predict metastatic involvement and tumor aggressiveness in this single-center retrospective study.

**Methods:**

This retrospective, single-center study enrolled 48 patients with suspected PCa (39 confirmed PCa, 9 benign conditions) who underwent [^99^ᵐTc]Tc-PSMA-I&S SPECT/CT between September 2022 and November 2023. Imaging was performed 4 h post-injection of 0.74 GBq [^99^ᵐTc]Tc-PSMA-I&S. Systematic prostate biopsy or surgical specimens served as the reference standard. Maximum standardized uptake values (SUVmax) were quantified in regions of enhanced prostatic uptake using Q.Volumetrix software. Correlations between SUVmax and clinicopathological parameters were analyzed using receiver operating characteristic (ROC) curves.

**Results:**

[^99^ᵐTc]Tc-PSMA-I&S SPECT/CT achieved 100% sensitivity, 77.78% specificity, and 95.83% accuracy. SUVmax correlated significantly with Gleason score, PSA levels, risk stratification, and metastatic status. Median SUVmax was significantly elevated in patients with PSA > 20 ng/mL versus ≤20 ng/mL (13.20 vs. 6.68; *p* = 0.013) and Gleason score >7 versus ≤7 (13.60 vs. 6.75; *p* = 0.006). High-risk and metastatic cohorts demonstrated significantly higher SUVmax values (*p* = 0.010 and *p* = 0.023, respectively). For high-risk PCa prediction, optimal SUVmax cutoff was ≥10.85 (AUC = 0.84; sensitivity = 100%, specificity = 58%). For metastatic PCa detection, optimal cutoff was SUVmax ≥14.45 (AUC = 0.73; sensitivity = 92%, specificity = 50%).

**Conclusion:**

[^99^ᵐTc]Tc-PSMA-I&S SPECT/CT demonstrates excellent diagnostic performance for PCa detection. SUVmax serves as a robust predictor for risk stratification and metastatic potential assessment.

## Introduction

Prostate cancer (PCa) represents the second most prevalent malignant neoplasm among men worldwide, constituting approximately 7.3% of all cancer cases and ranking as the fifth leading cause of cancer-related mortality in males (1). In China, the incidence of PCa has increased significantly, surpassing both bladder and kidney cancers to become the most common malignant tumor in the male urogenital system (2). The biological behavior of PCa varies considerably based on its malignancy grade, which directly influences treatment strategies and prognosis.

Early-stage PCa demonstrates excellent outcomes, with nearly 100% five-year survival rates through surgical intervention and androgen deprivation therapy. However, metastatic PCa presents a markedly different prognosis, with five-year survival rates ranging from 36 to 54% and a median survival time of approximately 42 months (3). Thus, early diagnosis and accurate grading of PCa are of great significance for formulating therapeutic strategies and improving prognosis. Currently, multiparametric magnetic resonance imaging (mpMRI) is the most widely utilized imaging technique for the diagnosis of PCa. mpMRI offers high-resolution soft tissue imaging, enabling a detailed assessment of the anatomical structure of the prostate, precise localization of primary PCa lesions, and visualization of the involvement of pelvic lymph nodes and bones. However, studies indicate that mpMRI exhibits a sensitivity of up to 96% in detecting PCa, whereas its specificity ranges from 36 to 58%, reflecting a relatively high false-positive rate (4, 5). At the same time, mpMRI’s inability to provide whole-body imaging in a single examination limits its utility for comprehensive staging (6). Bone scintigraphy (BS), a sensitive and cost-effective imaging modality for detecting PCa bone metastases, is constrained by limited specificity due to frequent false-positive findings caused by benign bone conditions (7). Therefore, the exploration of precise morphological and functional characterization is crucial for the clinical management of PCa.

Prostate-specific membrane antigen (PSMA), a Type II transmembrane glycoprotein predominantly expressed in prostatic tissues, demonstrates upregulated expression correlated with malignancy grade and metastatic progression (8). PSMA has emerged as a crucial target for both diagnostic imaging and radionuclide therapy in PCa. Extensive research has demonstrated the significant value of [68Ga]- and [18F]-labeled PSMA positron emission tomography/computed tomography (PET/CT) in diagnosis, treatment response evaluation, and patient follow-up (9–15). For instance, a systematic review published by Satapathy et al. (14) demonstrated the excellent sensitivity of [68Ga]Ga-PSMA-11 and [68Ga]Ga-PSMA-617 PET/CT for initial detection in patients with suspected PCa. Ergül et al. (15) demonstrated that [68Ga]Ga-PSMA-11 PET/CT is a highly effective imaging modality for the initial evaluation of newly diagnosed PCa, leading to significant changes in its staging compared to conventional imaging methods. In addition to the [68Ga]Ga-labeled PSMA radiotracers, Chikatamarla et al. (16) demonstrated comparable superior diagnostic accuracy for primary staging of PCa using [18F]F-labeled PSMA inhibitor ([18F]F-PSMA-1007) in their largest study. Notably, PSMA avidity exhibits a significant positive correlation with baseline serum total PSA levels and Gleason grade group classification, reinforcing its prognostic validity (10, 16). In addition, the maximum standardized uptake value (SUVmax) is the most commonly used semi-quantitative parameter in PET/CT, and a prospective study by Jiao et al. (17) demonstrated that the optimal SUVmax cut-off value for distinguishing clinically significant PCa from benign prostate disease was 5.30.

Despite these advantages, PET faces limitations in clinical applications due to the high costs associated with radiotracers and specialized equipment (18, 19). In contrast, single-photon emission computed tomography (SPECT)/CT is more globally accessible, providing a cost-effective alternative for PSMA-targeted imaging. Furthermore, recent advances in SPECT/CT fusion imaging, which provide both anatomical and functional information, have facilitated the development of single-photon-labeled PSMA tracers (20). In recent years, researchers have designed multiple [^99^ᵐTc]-labeled PSMA-targeted molecules for PCa detection (21–24).

Among these, [^99^ᵐTc]Tc-PSMA-I&S, a PSMA-targeted compound introduced by Robu et al. (25) in 2016, was initially developed for radioguided surgery. This radiotracer is characterized by slow systemic clearance, enabling prolonged retention in tumor cells. Over 21 h post-injection, the lesion-to-background contrast progressively increases, making it particularly suitable for intraoperative detection and excision of PSMA-positive lymph node metastases. This distinctive characteristic may provide superior lesion-to-background contrast in delayed imaging, thereby potentially offering diagnostic advantages over other SPECT-based PSMA tracers, including [^99^ᵐTc]Tc-MIP-1404 and [^99^ᵐTc]Tc-HYNIC-PSMA. Owing to its stable and reproducible labeling process, [^99^ᵐTc]Tc-PSMA-I&S demonstrates potential for SPECT imaging (25). Furthermore, advancements in SPECT/CT image reconstruction algorithms, photon attenuation correction, and scatter correction techniques have significantly enhanced the clinical utility of quantitative SPECT/CT.

Therefore, this study aims to investigate the clinical utility of [^99^ᵐTc]Tc-PSMA-I&S SPECT/CT in the diagnosis of PCa, prediction of tumor metastasis, and assessment of malignancy grade.

## Methods

This study adhered to the guiding principles of the Declaration of Helsinki and was approved by the Ethics Committee of Panzhihua Central Hospital (approval number: zhszxyykyll-2022-002). However, due to the retrospective nature of the study, written informed consent was waived.

### Patients

This study enrolled patients with suspected primary PCa who underwent [^99^ᵐTc]Tc-PSMA-I&S SPECT/CT scanning at the Department of Nuclear Medicine, Panzhihua Central Hospital, China, between September 1, 2022 and November 30, 2023. Inclusion criteria were defined as meeting any of the following conditions: (1) serum total prostate-specific antigen (tPSA) > 10 μg/L (normal reference range: <4 μg/L); (2) tPSA 4–10 μg/L with a free PSA (fPSA)/tPSA ratio <0.19; (3) digital rectal examination revealing suspicious prostatic nodules; (4) ultrasonographic or magnetic resonance imaging findings suggestive of malignancy. Exclusion criteria included: (1) incomplete clinical records or loss to follow-up; (2) prior PCa-directed interventions (including surgery, radiotherapy, chemotherapy, or endocrine therapy) before undergoing [^99^ᵐTc]Tc-PSMA-I&S SPECT/CT; (3) concurrent malignant neoplasms.

### Radiosynthesis and quality control of [99ᵐTc]Tc-PSMA-I&S

Fresh [^99^ᵐTc]NaTcO₄ eluate was procured from China Isotope & Radiation Corporation. PSMA-I&S precursors were synthesized by Nanchang Probe Technology Co., Ltd. (China), with sterile pyrogen-free lyophilized kits prepared by Shanghai Jiabiao Biotechnology Co., Ltd. (China). [^99^ᵐTc]Tc-PSMA-I&S was synthesized following established protocols (25): (1) One vial of PSMA-I&S precursor was reconstituted with 1.0 mL freshly eluted [^99^ᵐTc]NaTcO₄ (1.48–2.96 GBq [40–80 mCi]); (2) The mixture was vortexed for 30 s to ensure homogeneity; (3) Radiolabeling proceeded at 100 °C under atmospheric pressure for 20 min with intermittent agitation; (4) The reaction mixture was cooled to 40 °C, filtered through a 0.22 μm hydrophilic PVDF membrane, and diluted with sterile saline to appropriate radioactivity concentration. Quality control analyses confirmed >95% radiochemical purity and labeling efficiency.

### [^99^ᵐTc]Tc-PSMA-I&S SPECT/CT acquisition

All patients underwent whole-body planar scintigraphy and SPECT/CT imaging 4–6 h after intravenous administration of 0.74 GBq (20 mCi) of [^99^ᵐTc]Tc-PSMA-I&S. Patient identification was verified prior to imaging. The injection time and pre- and post-injection syringe activities were measured and recorded to calculate the actual administered dose. Vital signs (respiratory rate, heart rate, and blood pressure) and non-specific symptoms (nausea, vomiting, headache, dizziness, rash, or pruritus) were monitored at 10 min before injection and at 10 and 60 min post-injection, with symptomatic treatment provided as necessary. Patients were instructed to ingest at least 1,000 mL of water within 4 h post-injection and to void frequently to promote hydration and tracer excretion, thereby reducing background activity and enhancing image quality. Additionally, patients were asked to void 5 min before imaging to prevent bladder activity from obscuring prostate bed lesions and pelvic lymph node metastases. In cases of dysuria or urinary retention, catheterization was performed prior to scanning.

Imaging utilized a Discovery NM/CT 670 SPECT/CT system (GE Healthcare) with dual-detector configuration. Whole-body anterior–posterior projections were acquired at 16.0 cm/min scan speed (140.5 keV photopeak, ±7.5% energy window, 256 × 1,024 matrix). SPECT/CT acquisition followed a standardized protocol: initial low-dose CT (120 kV tube potential, 512 × 512 matrix, 2.5 mm slice thickness, iterative reconstruction with attenuation correction) preceded SPECT acquisition.

### SPECT/CT image analysis and validation

Image analysis was performed on a Xeleris workstation version 4 DR (GE Healthcare). Two nuclear medicine physicians with more than 10 years of experience in reading SPECT/CT images jointly reviewed SPECT/CT images. When the two physicians disagreed, a consensus was reached through discussion. After excluding physiological or obvious nonprostate cancer-related uptake, the foci where [^99^ᵐTc]Tc-PSMA-I&S uptake was higher than that of the surrounding normal tissue were defined as positive.

Quantitative analysis was performed using Q.Volutrix software on the same workstation (26). The analysis required input of patient anthropometric measurements (height and weight), radioisotope specifications (^99^ᵐTc), precise injected dose, timing of drug administration, image acquisition time, and camera sensitivity. SUVmax values (g/mL) were calculated using automated volume-of-interest (VOI) delineation. In cases with multiple prostatic lesions, the highest SUVmax was selected as the representative value.

### Standard reference for imaging results

SPECT/CT findings were categorized as (a) primary tumor or (b) extraprostatic metastases (including lymph node, osseous, and visceral organ involvement). Prostate needle biopsies were performed in all participants. For biopsy-positive cases meeting surgical criteria, radical prostatectomy was performed, with pathological confirmation based on surgical specimens. In biopsy-positive patients deemed ineligible for surgery, pathological diagnosis relied solely on biopsy specimens. For patients with negative biopsy results but strong clinical suspicion of PCa, serial monitoring of serum PSA levels combined with imaging findings (mpMRI and [^99^ᵐTc]Tc-PSMA SPECT/CT) was conducted over 3–6 months. Absence of disease progression during surveillance allowed exclusion of PCa diagnosis, whereas evident progression warranted repeat biopsy (27). While all primary tumors underwent histopathological confirmation via biopsy or surgical specimens, extraprostatic metastatic lesions could not be histologically verified in all cases. Metastatic disease was therefore confirmed through: (1) concordant findings on multimodal imaging (bone scintigraphy, MRI); (2) serial PSA monitoring over 6-month follow-up; (3) response to systemic therapy; and (4) imaging surveillance as per established clinical criteria (17, 28). This approach, while clinically reasonable, may introduce uncertainty in sensitivity/specificity estimates for metastatic detection.

### Statistical analysis

All statistical analyses were conducted using R statistical software (version 4.2.0). Categorical variables were expressed as counts (percentages), while continuous variables were summarized as mean ± standard deviation (SD) for normally distributed data or median with interquartile range (IQR) for nonparametric distributions. Correlations between SPECT/CT-derived SUVmax in primary PCa foci and clinical variables were assessed using Spearman’s rank correlation analysis. Intergroup comparisons were performed using the nonparametric Wilcoxon-Mann–Whitney U test with two-tailed significance testing. Using histopathology as the reference standard, diagnostic performance metrics [sensitivity, specificity, accuracy, positive predictive value (PPV), and negative predictive value (NPV)] were calculated for [^99^ᵐTc]Tc-PSMA-I&S SPECT/CT imaging. The discriminative capacity of SUVmax for risk stratification and metastatic detection was evaluated through receiver operating characteristic (ROC) curve analysis, with quantification of the area under the curve (AUC) and 95% confidence intervals (95% CI). Optimal SUVmax thresholds were identified through Youden index maximization. Statistical significance was defined as *p* < 0.05.

## Results

### Patient characteristics

**Figure 1:**
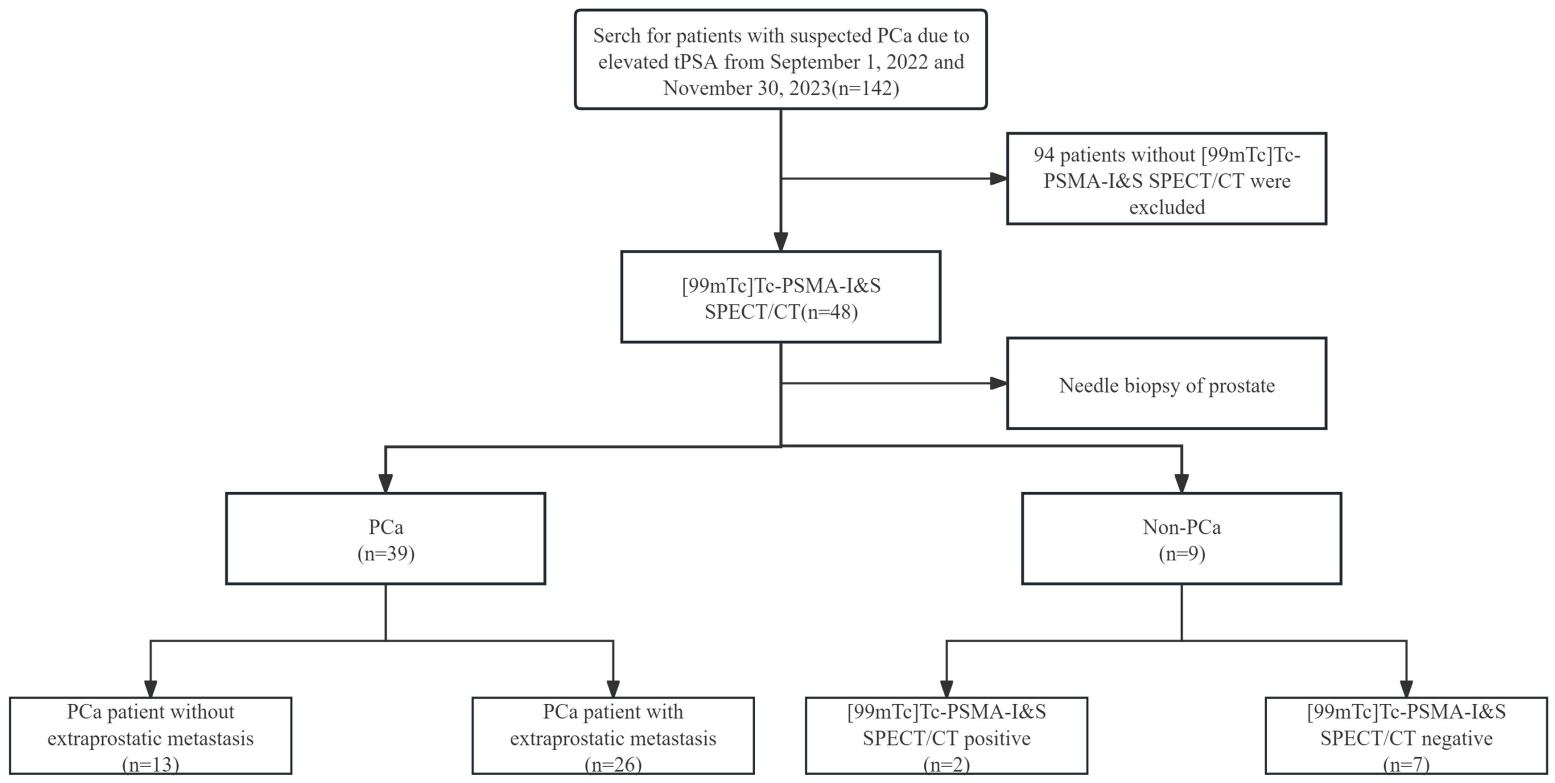
Flowchart of participant selection in the study.

This retrospective study enrolled 48 patients undergoing [^99^ᵐTc]Tc-PSMA-I&S SPECT/CT examinations. Figure 1 illustrates the participant inclusion flowchart, with all procedures completed without adverse events. Table 1 summarizes the demographic and clinical characteristics of the cohort. The median age at [^99^ᵐTc]Tc-PSMA-I&S SPECT/CT imaging was 75 years (range: 47–96 years). The median pre-imaging PSA level was 43.63 ng/mL (range: 11.00–100.00 ng/mL), with 15 patients (31.3%) having PSA ≤ 20 μg/L and 33 patients (68.8%) having PSA > 20 μg/L. All patients underwent biopsy or surgical pathological examination. Among the 48 patients, 39 were diagnosed with PCa, while 9 were non-PCa cases (7 with prostatic hyperplasia and 2 with prostatic hyperplasia accompanied by chronic inflammatory lesions). In the PCa cohort, Gleason score distribution was as follows: score 6 (*n* = 4), 7 (*n* = 9), 8 (*n* = 11), 9 (*n* = 10), and 10 (*n* = 5). Based on NCCN guidelines (5), patients were stratified into low-intermediate risk (requiring all criteria: PSA ≤ 20 μg/L, Gleason score 6–7, and cT1–cT2c) and high risk (meeting any criterion: PSA > 20 μg/L, Gleason score 8–10, or ≥cT3). Six patients (15.4%) were classified as low-intermediate risk, while 33 patients (84.6%) were classified as high risk. Metastatic disease was identified in 26 PCa patients (66.7%), with lymph nodes being the predominant site of extraprostatic spread.

**Table 1:** Demographic and clinical characteristics of the 48 study participants.

Characteristic	Value
Age at SPECT/CT (years)	
Median (range)	75.00 (47, 96)
Mean ± SD	74.17 ± 9.77
tPSA level (ng/mL)	
Median (range)	43.63 (11.00, 100.00)
Mean ± SD	55.30 ± 38.83
≤20	15 (31.25)
>20	33 (68.75)
Pathology of biopsies	
Prostate cancer patients	39 (81.30%)
Non-prostate cancer patients	9(18.80%)
Gleason score	
6	4/39(10.30%)
7	9/39(23.10%)
8	11/39(28.20%)
9	10/39(25.60%)
10	5/39(12.80%)
Risk group	
Low-intermediate risk	6/39 (15.40%)
High risk	33/39 (84.60%)
SUVmax of prostate region	
Median (range)	10.30(2.58, 73.20)
Mean ± SD	18.37 ± 19.09
Metastasis on SPECT/CT imaging	
Non-metastatic patients (%)	13 /39 (33.30%)
Metastatic patients (%)	26/39 (66.70%)
Site of extraprostatic metastases	
Lymph node metastases	22/39(56.40%)
Bone metastases	19/39(48.70%)
Visceral metastases	1/39(2.60%)

### Visual analysis of [^99^ᵐTc]Tc-PSMA-I&S SPECT/CT imaging

**Figure 2:**
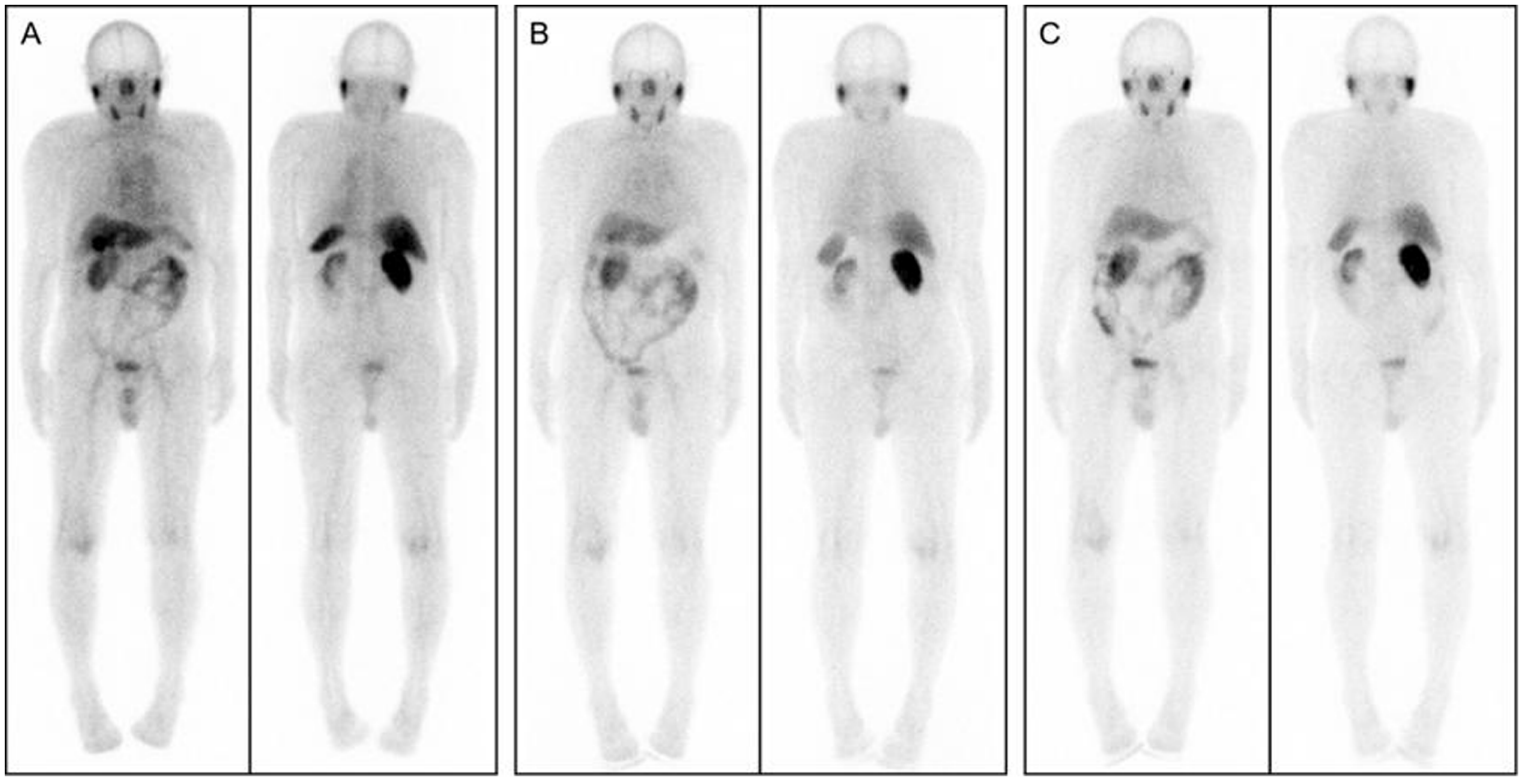
Whole-body SPECT images acquired at 1, 4, and 6 h after [^99^ᵐTc]Tc-PSMA-I&S injection: **(A)** 1-h post-injection; **(B)** 4-h post-injection; **(C)** 6-h post-injection.

As illustrated in Figure 2, whole-body SPECT imaging was performed at 1, 4, and 6 h post-injection of [^99^ᵐTc]Tc-PSMA-I&S. Visual assessment revealed that 1-h (A) images exhibited greater physiological tracer retention at various anatomical sites compared to 4-h (B) and 6-h (C) images. However, 4-h and 6-h images demonstrated comparable quality in terms of resolution and lesion visualization.

Considering clinical practicality and patient compliance, 4 h post-injection was selected as the optimal imaging timepoint. In 4-h images, the physiological tracer distribution manifested three distinct uptake levels:

- High uptake regions: bilateral parotid glands, submandibular glands, kidneys, and bladder.
- Moderate uptake regions: liver, spleen, and intestinal tract.
- Low uptake regions: heart, lungs, thyroid, muscles, bones, and brain tissue.

**Figure 3:**
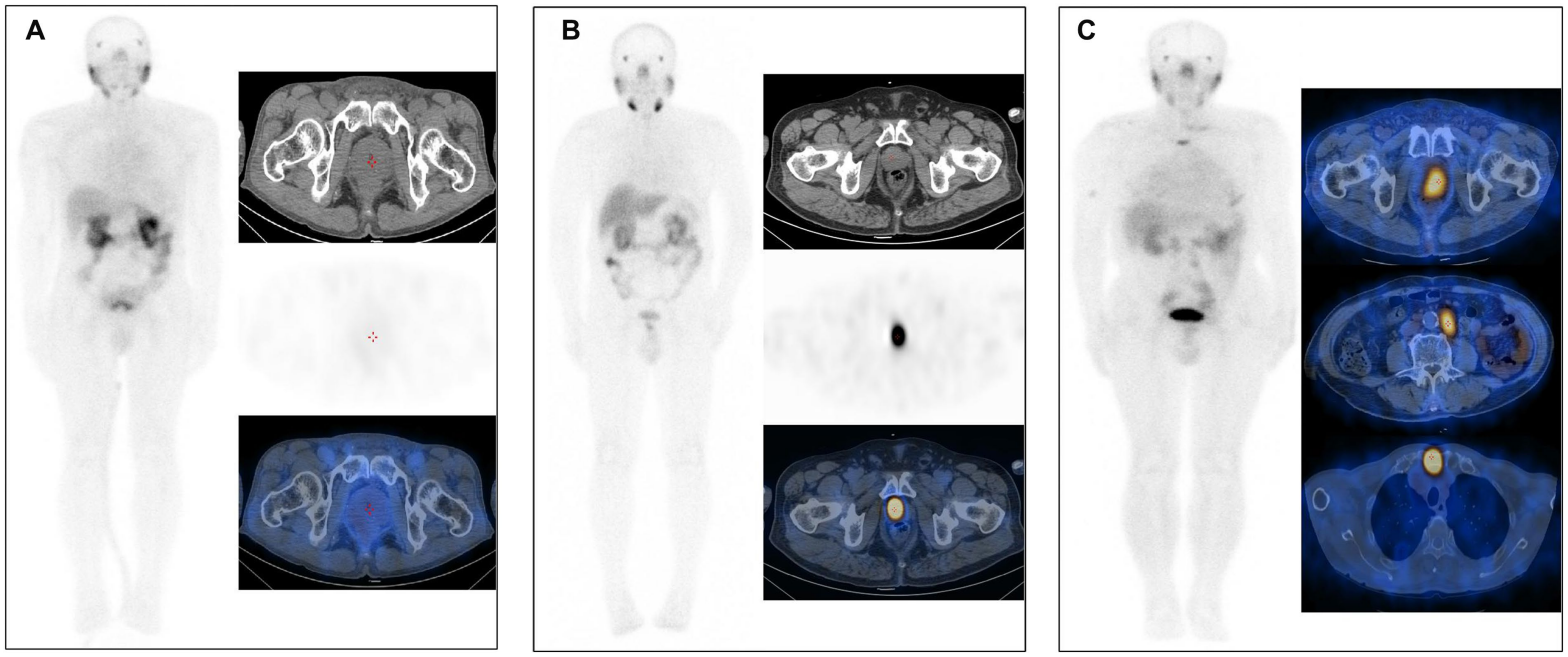
**(A)** [^99^ᵐTc]Tc-PSMA-I&S SPECT/CT imaging of a patient with benign prostatic hyperplasia (73 years old, tPSA 11.00 ng/mL). No significant abnormalities were observed on either whole-body planar scanning (anterior view) or axial SPECT/CT images. **(B)** [^99^ᵐTc]Tc-PSMA-I&S SPECT/CT imaging of a PCa patient [75 years old, Gleason score 7 (4 + 3), tPSA 29.01 ng/mL]. The whole-body planar scan (anterior view) and axial SPECT/CT fusion images demonstrated a primary tumor within the prostate. **(C)** [^99^ᵐTc]Tc-PSMA-I&S SPECT/CT imaging of a PCa patient [85 years old, Gleason score 9 (4 + 5), tPSA 100 ng/mL]. The whole-body planar scan (anterior view) and axial SPECT/CT fusion images revealed a primary tumor within the prostate, multiple lymph node metastases, and bone metastases.

The visual analysis results of [^99^ᵐTc]Tc-PSMA-I&S SPECT/CT imaging for all 48 patients are summarized in Table 2. Among the 9 non-PCa patients, 7 demonstrated negative findings with no areas of enhanced radiotracer uptake throughout the body (a representative case is shown in Figure 3A), while 2 patients exhibited focal increased tracer uptake in the prostate (SUVmax values of 3.26 and 3.73, respectively) without abnormal accumulation in extra-prostatic areas. Of the 39 patients with PCa, 13 demonstrated increased uptake confined to the prostate without evidence of metastatic lesions (illustrated in Figure 3B), and 26 showed increased uptake in both primary prostatic lesions and metastatic sites (representative images in Figure 3C). Based on visual analysis, [^99^ᵐTc]Tc-PSMA-I&S SPECT/CT achieved a sensitivity of 100% (39/39), specificity of 77.8% (7/9), positive predictive value of 95.1% (39/41), negative predictive value of 100% (7/7), and overall accuracy of 95.8% (46/48) for PCa detection. Visual analysis alone could not reliably differentiate between PCa and non-PCa patients exhibiting isolated prostatic uptake; however, semi-quantitative analysis revealed that PCa patients generally demonstrated higher SUVmax values (mean SUVmax: 18.37 ± 19.09) compared to the two false-positive non-PCa cases. Differences in Primary Lesion SUVmax Values among patient subgroups.

**Table 2:** Visual analysis of [^99^ᵐTc]Tc-PSMA-I&S SPECT/CT imaging in diagnosis of prostate cancer.

Pathological diagnosis	[^99m^Tc]Tc-PSMA-I&S SPECT/CT	
	Positive	Negative
Non-prostate cancer	2	7
Prostate cancer	39	0
Total	41	7

**Figure 4:**
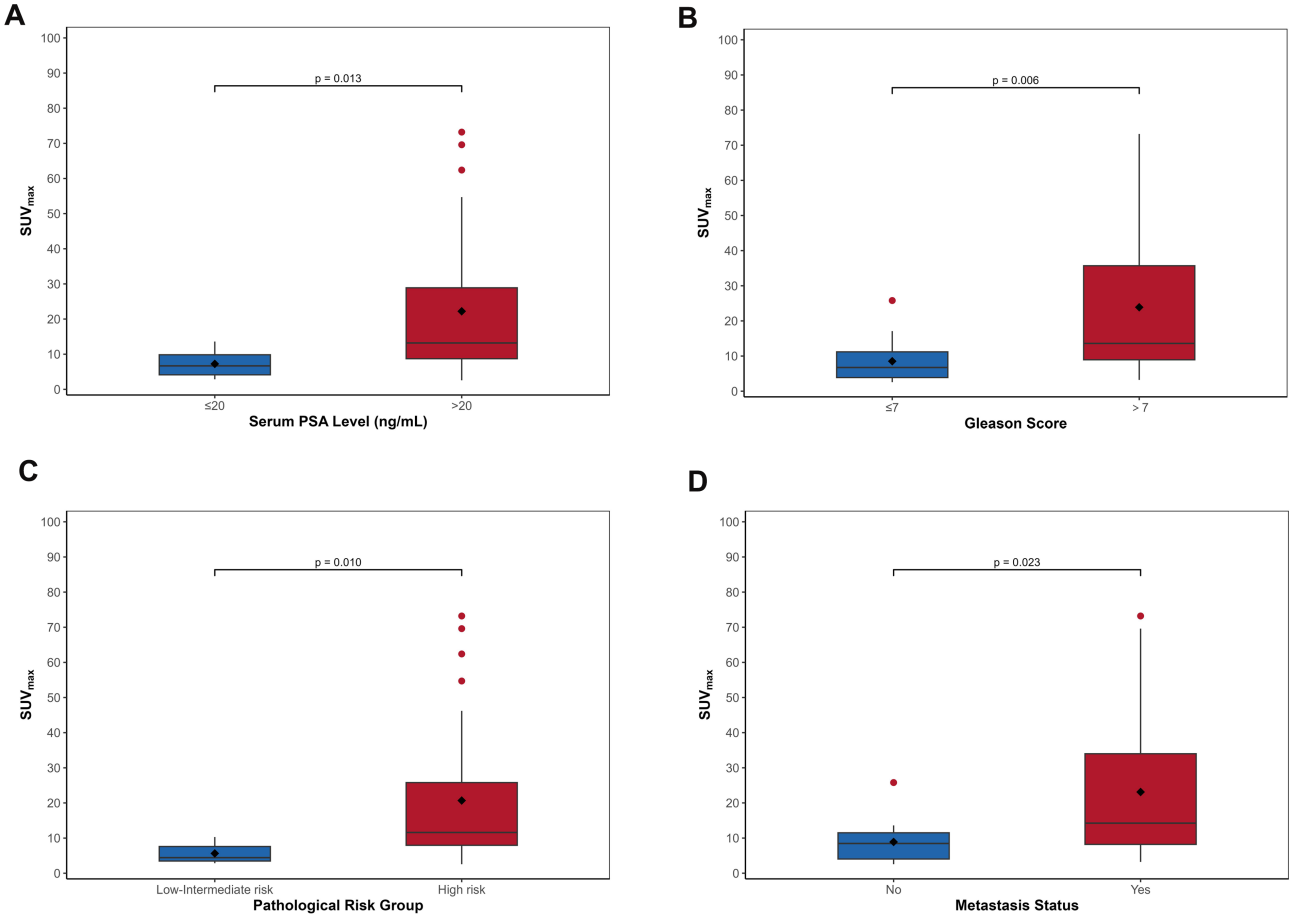
The boxplots demonstrate progressive increases in SUVmax of primary PCa correlating with elevated tPSA levels **(A)**, higher Gleason scores **(B)**, advanced risk stratification **(C)**, and the presence of metastatic disease **(D)**.

Given that PSMA uptake was observed in only 2 non-PCa patients, with negligible uptake in the remaining visually negative non-PCa patients, SUVmax analysis was not performed for the non-PCa group. In the PCa cohort (*n* = 39), primary lesions demonstrated a median SUVmax of 10.30 (range: 2.58–73.20). The correlation between SUVmax and PSA levels is illustrated in Figure 4. Patients with tPSA exceeding 20 ng/mL exhibited significantly higher median SUVmax values [13.20 (8.72–28.90)] compared to those with tPSA ≤20 ng/mL [6.68 (4.13–9.84); *p* = 0.013] (Figure 4A). Similarly, cases with Gleason scores >7 showed markedly elevated SUVmax values [median: 13.60 (8.94–35.70)] versus those with scores ≤7 [median: 6.75 (3.88–11.20); *p* = 0.006] (Figure 4B). Additionally, high-risk and metastatic subgroups demonstrated significantly higher SUVmax values compared to their low/intermediate-risk (Figure 4C) and non-metastatic counterparts (Figure 4D) (*p* = 0.010 and *p* = 0.023, respectively). Spearman correlation analyses revealed significant positive associations between SUVmax and multiple clinical parameters, including Gleason score (rs = 0.542), tPSA (rs = 0.472), risk stratification (rs = 0.423), and metastatic status (rs = 0.372) (all *p* < 0.05).

### The predictive value of primary tumor SUVmax for risk stratification and metastasis

**Figure 5:**
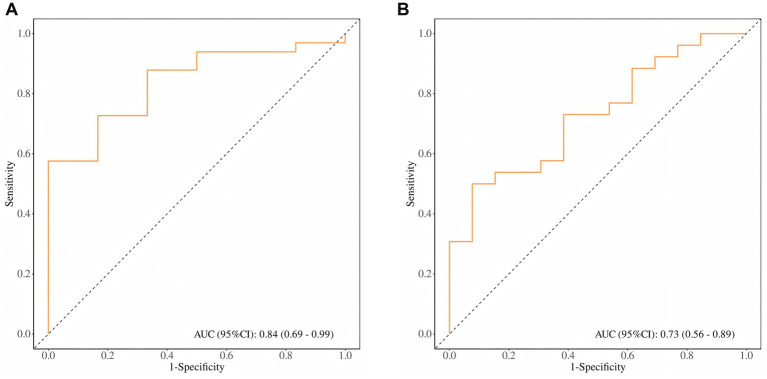
**(A)** ROC curve of SUVmax for PCa patient risk stratification; **(B)** ROC curve of SUVmax for PCa distant metastasis risk prediction.

ROC curve analysis evaluated SUVmax’s predictive capacity for risk stratification and metastasis. For risk stratification, SUVmax demonstrated robust performance (AUC = 0.84, 95% CI: 0.69–0.99), achieving 100% sensitivity and 58% specificity at an optimal cutoff of 10.85 (Figure 5A). For metastatic risk prediction, SUVmax showed moderate efficacy (AUC = 0.73, 95% CI: 0.56–0.89), with 92% sensitivity and 50% specificity at a cutoff of 14.45 (Figure 5B).

## Discussion

PCa is one of the most prevalent malignancies among men. Early diagnosis and accurate risk stratification are critical for optimizing therapeutic strategies and improving patient prognosis (27). PSMA-targeted PET imaging has emerged as the gold standard for prostate cancer imaging, enabling highly sensitive early detection of subcentimeter primary malignancies, precise lymph node staging and metastatic assessment, recurrence detection, and therapeutic response evaluation. PSMA PET imaging has been incorporated into major clinical management guidelines, including those of the National Comprehensive Cancer Network, the European Society for Medical Oncology, and the European Association of Urology (29–31). While PET/CT with [^68^Ga]Ga-PSMA or [^18^F]F-PSMA tracers has demonstrated exceptional efficacy in primary PCa detection (32), its widespread clinical implementation is constrained by high operational costs. In contrast, SPECT/CT offers broader accessibility and cost-effectiveness, positioning it as a viable alternative for PSMA-targeted imaging. Among recently developed [^99^ᵐTc]Tc-labeled PSMA probes, [^99^ᵐTc]Tc-PSMA-I&S—originally designed for radioguided surgery—has emerged as a promising tracer for PCa imaging. Analyzed the application value of [^99^ᵐTc]Tc-PSMA-I&S SPECT/CT in the diagnosis, metastasis prediction, and tumor invasion assessment of PCa.

Based on comprehensive analysis of image quality, clinical practicality, and patient compliance, we identified 4 h post-injection as the optimal imaging timepoint for [^99^ᵐTc]Tc-PSMA-I&S. Biodistribution analysis revealed characteristic tracer uptake patterns: high uptake in bilateral parotid glands, submandibular glands, kidneys, and bladder; moderate uptake in the liver, spleen, and small intestine; and low uptake in the heart, lungs, thyroid, muscles, bones, and brain tissues. These findings indicate predominant tracer excretion via the urinary and hepatobiliary systems, with minimal physiological uptake in non-target tissues. The absence of adverse events further underscores the safety and stability of [^99^ᵐTc]Tc-PSMA-I&S for clinical use.

In the present study, [^99^ᵐTc]Tc-PSMA-I&S SPECT/CT exhibited excellent diagnostic performance for primary PCa, with sensitivity, specificity, positive predictive value, negative predictive value, and accuracy of 100, 77.28, 95.12, 100, and 95.83%, respectively. These results align with those reported by Farkas et al. (33), who demonstrated comparable diagnostic metrics (sensitivity 86%, specificity 100%, positive predictive value 100%, negative predictive value 83%, and accuracy 92%). Werner et al. (22) similarly demonstrated high sensitivity (92%) for [^99^ᵐTc]Tc-PSMA-I&S SPECT/CT in detecting primary PCa. These findings are also consistent with previous studies by Wang et al. (34) and Goffin et al. (35), who reported detection rates of 100 and 94% for primary tumors in 31 and 104 PCa patients, respectively, using other [^99^ᵐTc]Tc-labeled PSMA radiotracers (MIP-1404 and HYNIC-PSMA) for initial staging. Regarding PSMA-targeted PET tracers, Basha et al. (36) reported a detection rate of 96% using [68Ga]Ga-PSMA-11 PET/CT in 173 primary PCa patients. Collectively, these results underscore the promising potential and clinical utility of [^99^ᵐTc]Tc-PSMA-I&S for primary tumor detection in early PCa diagnosis.

Despite high diagnostic accuracy, PSMA-targeted imaging is not devoid of false-positive findings. In our study, [^99^ᵐTc]Tc-PSMA-I&S SPECT/CT imaging yielded positive results in 41 cases, with 2 cases being false positives. Previous studies have shown that false-positive PSMA PET/CT findings in prostatic tissue can be attributed to various benign conditions, including hyperplasia, inflammatory processes, and glandular fibrosis secondary to repeated biopsies (37). These documented factors may explain the two false-positive cases observed in our study.

PSMA expression levels in PCa demonstrate significant positive correlations with tumor stage, Gleason score, and pre-treatment tPSA levels, with elevated PSMA expression typically indicating increased malignancy (38, 39). Studies have established that abnormally elevated PSMA expression serves as a predictive indicator for PCa recurrence and metastasis (40). Previous studies using both PET ([^68^Ga]Ga−/[^18^F]F-PSMA) and SPECT ([^99^ᵐTc]Tc-HYNIC-PSMA) modalities have demonstrated significant associations between SUVmax and clinical parameters including tPSA and Gleason score (10, 34, 41). Consistent with these findings, our study revealed similar correlations through semi-quantitative analysis of [^99^ᵐTc]Tc-PSMA-I&S SPECT/CT imaging, where SUVmax showed positive associations with tPSA, Gleason score, risk stratification, and disease advancement. Importantly, patients with tPSA ≤20 ng/mL, Gleason score ≤7, low-intermediate risk classification, or non-metastatic disease exhibited significantly lower SUVmax values than their counterparts, underscoring SUVmax’s utility in risk stratification.

Early detection of metastatic disease in PCa patients is crucial for developing effective treatment strategies and avoiding unnecessary major surgical interventions. Multiple studies have demonstrated that PSMA PET/CT exhibits superior efficacy in detecting distant metastases in primary PCa patients compared to conventional imaging methods. This modality accurately reflects the degree of malignancy and disease staging while reducing the need for repeated examinations and invasive biopsies (42–45). Our findings demonstrate that [^99^ᵐTc]Tc-PSMA-I&S SPECT/CT serves as a valuable and reliable tool for detecting advanced disease. In our patient cohort, this imaging modality successfully identified metastatic lesions across multiple sites: lymph node involvement in 56.4% of patients, bone metastases in 48.7%, and visceral metastases in 2.6%. Notably, lymph nodes emerged as the predominant site of extra-prostatic spread.

Direct comparative analysis with Ga-PSMA PET/CT was not conducted in our study, as this imaging modality was not available at our institution during the study period. However, existing literature demonstrates that Tc-PSMA imaging exhibits comparable diagnostic performance to PSMA PET/CT in metastatic evaluation. Albalooshi et al. (18) systematically compared the diagnostic efficacy of Tc-PSMA versus Ga-PSMA PET/CT in 28 patients with prostate cancer (PCa). The investigators reported no statistically significant differences between the two imaging modalities in detecting lymph node and distant disease (*p* > 0.05). Similarly, Fallahi et al. (46) conducted a comparative study involving 22 PCa patients and demonstrated equivalent detection rates of lymph node and distant metastases with ^99^ᵐTc-PSMA SPECT/CT compared to Ga-PSMA PET/CT imaging. Furthermore, Singh et al. (47) indicated that whole-body Tc-PSMA combined with regional SPECT/CT represents a viable alternative to Ga-PSMA PET for detecting advanced metastatic prostate cancer and evaluating therapeutic response to PSMA-based radioligand therapy. Concurrently, patient selection for Lu-PSMA radioligand therapy (RLT) necessitates sequential PSMA imaging followed by therapeutic monitoring, imposing a substantial economic burden on these patients. Within this clinical context, SPECT/CT imaging, characterized by lower costs and greater accessibility, represents a practical alternative that may improve patient compliance and enhance adherence to standardized monitoring protocols (48, 49).

A distant metastasis risk prediction model based on SUVmax can serve as a factor for evaluating distant metastases. During visual assessment, extra-prostatic distant lesions showed heterogeneity and false-positive rates. When pathological results are unavailable, this may influence treatment selection. Based on our results, an optimal SUVmax value of 14.45 with 92% sensitivity may provide reference for distant metastasis diagnosis. Furthermore, the prediction model based on SUVmax demonstrated an area under the ROC curve of 0.84, effectively distinguishing high-risk PCa patients. Bjurlin et al. (50) proposed that prediction models for high-risk PCa should primarily have high sensitivity to screen for patients with higher malignancy and metastatic risk while maintaining good specificity. Our study shows that the [^99^ᵐTc]Tc-PSMA-I&S SPECT/CT prediction model achieved sensitivity and specificity of 100 and 58%, respectively, (cutoff value 10.85), indicating its predictive value in PCa risk stratification.

However, this study has certain limitations. First, the retrospective, single-center design coupled with a limited sample size (*n* = 48, including 39 prostate cancer cases) constrains statistical power and generalizability to some extent. Future validation through larger, multicenter prospective studies will be necessary. Second, while the composite reference standard used for metastatic disease validation is clinically appropriate, it introduces uncertainty in specificity assessment since not all lesions underwent histopathological confirmation. Third, the PSMA molecular probe utilized in this study undergoes urinary excretion, which may attenuate metastatic signals near the bladder and kidneys, potentially masking tumor lesions in these regions. Furthermore, our study lacks direct comparison with [68Ga]Ga-PSMA PET/CT, which represents the current imaging gold standard for PSMA-targeted imaging. Future prospective studies incorporating head-to-head comparisons between these imaging modalities will provide valuable insights into their relative diagnostic performance and clinical utility.

## Conclusion

In conclusion, our study demonstrates that Tc-PSMA-I&S SPECT/CT imaging constitutes a safe, reliable, and non-invasive diagnostic approach for prostate cancer (PCa), demonstrating efficacy in risk stratification of primary PCa patients and detection of distant metastases. This holds significant value for guiding therapeutic strategies. In economically underdeveloped regions with limited PET/CT availability, it may be considered as an alternative or supplementary method to PSMA PET/CT.

## Data Availability

The raw data supporting the conclusions of this article will be made available by the authors, without undue reservation.
